# A Longitudinal Study on the Addictive Behaviors of General Population before and during the COVID-19 Pandemic in China

**DOI:** 10.3390/ijerph19105979

**Published:** 2022-05-14

**Authors:** Xiaoyu Wang, Zaifei Ma, Chunan Wang

**Affiliations:** 1 Institute of Population and Labor Economics, Chinese Academy of Social Sciences, Beijing 100006, China; wangxyecon@cass.org.cn; 2 School of Statistics, Renmin University of China, Beijing 100872, China; 2019202164@ruc.edu.cn; 3 School of Economics and Management, Beihang University, Beijing 100191, China; 4 Beihang Hangzhou Innovation Institute Yuhang, Hangzhou 310023, China; 5 MoE Key Laboratory of Complex System Analysis and Management Decision, Beijing 100191, China

**Keywords:** COVID-19 pandemic, addictive behavior, smoking, drinking, China

## Abstract

By using nationally representative longitudinal data, this study investigates the effects of the COVID-19 pandemic on the addictive behaviors (smoking and drinking) of the general population in China. From the China Family Panel Studies (CFPS) 2018 and 2020, we extract a sample of individuals over 16 years of age in China, consisting of 14,468 individuals and 28,936 observations. We decompose the sample into three age groups, that is, ages between 16 and 39, ages between 40 and 59 and ages above 60. The bootstrap method is used to estimate the confidence interval of the difference in the mean of addictive behaviors, and logit models are used in the regression analysis. Our results show that the COVID-19 pandemic reduces the smoking behavior of individuals above 40 years of age, and that it reduces the drinking behavior of individuals above 16 years of age. However, the pandemic increases the smoking behavior of individuals between 16 and 39 years of age. These results may be closely related to the characteristics of COVID-19 (that is, a respiratory system disease), the working and economic pressures of young Chinese and the role of drinking alcohol in building and maintaining social networks in China.

## 1. Introduction

Many studies have investigated the effects of the COVID-19 pandemic on addictive behaviors, including smoking and drinking behaviors [1,2,3,4,5,6]. There is evidence that the COVID-19 pandemic may have reduced tobacco consumption due to perceived infection risks and decreased social smoking under stay-at-home orders [7,8], while there is also evidence that daily life stresses, the removal of barriers to smoking due to the change in workplace and feelings of loneliness and boredom during the COVID-19 pandemic may have increased cigarette use [9,10,11]. However, as a result of a number of determinants of smoking behavior, such as the above-mentioned stresses and the perceived infection risks, most studies find heterogeneous effects of the COVID-19 pandemic on tobacco consumption in a sample or population, that is, a proportion of individuals with increased use of tobacco, a proportion of individuals with constant use and a proportion of individuals with decreased use [12,13,14,15,16].

In terms of drinking behavior, it is found that alcohol consumption may have decreased for men, while it was stable for women [17]. Nevertheless, more evidence of increased alcohol consumption is found [18,19,20,21]. A cross-sectional study in the USA shows that the majority of respondents reported increased alcohol consumption, while only 13% of respondents reported decreased alcohol use. The most common reasons for increased alcohol consumption include increased stress, alcohol availability and boredom during the COVID-19 pandemic [18]. In a study conducted in Norway, more than half of individuals in the sample reported binge drinking behavior during the COVID-19 lockdown, and such a phenomenon is closely related to economic pressure and staying at home [19]. An online survey provides evidence that compared to regions with no restrictions, individuals in regions with COVID-19 lockdowns reported binge drinking behavior more and more over time [20]. In addition, there is also evidence that although the total alcohol consumption changed slightly, the proportion of binge drinking behavior increased significantly [21]. Further, analogously to smoking behavior, a number of studies demonstrate heterogeneous effects of the COVID-19 pandemic on alcohol consumption [22,23,24].

From the perspective of the research scope, the majority of studies focus on the effects of the COVID-19 pandemic on specific groups of individuals or populations in local areas or countries. For example, some studies examine the effects on the smoking and drinking behaviors of university students in the Netherlands, Poland, Czech Republic, Slovak Republic, the USA, Portugal, and France [25,26,27,28,29,30,31,32]. Some other studies investigate the effects of the COVID-19 pandemic on the smoking and drinking behaviors in England, the UK, Saudi Arabia, Mexico, eight European countries (Czech Republic, Denmark, Finland, Germany, Norway, Poland, Spain, and the UK), and Latin America and the Caribbean [33,34,35,36,37,38].

As for China, to the best of our knowledge, although there are a number of studies discussing the effects of the COVID-19 pandemic on individuals’ mental health [39,40,41,42,43], studies regarding the effects on addictive behaviors (smoking and drinking) are scarce [44]. Therefore, using nationally representative high-quality longitudinal data, this paper aims to contribute to the literature by investigating how the COVID-19 pandemic affects the smoking and drinking behaviors of the general population in China.

As shown by a large number of studies, the individuals’ mental health deteriorates during the COVID-19 pandemic [45,46,47,48,49,50,51,52,53,54,55,56,57,58,59]. The higher degree of mental health problems manifested as, for example, stress, anxiety and depression, may result in heavier tobacco and alcohol use during the pandemic. However, tobacco and alcohol consumption, especially drinking alcohol, play an important role in building and maintaining social networks in many regions of China. The lockdowns and working/staying at home during the COVID-19 pandemic reduce social activities substantially and thus may decrease the use of tobacco and alcohol. In addition, individuals of different ages may have distinct perceptions of infection risks and different needs for social activities. Therefore, without empirical analysis, it is hard to estimate the effects of the COVID-19 pandemic on the smoking and drinking behaviors of the general population in China.

## 2. Materials and Methods

### 2.1. Data

The China Family Panel Studies (CFPS) is a nationally representative longitudinal survey launched in 2010 and implemented by the Institute of Social Science Survey of Peking University. CFPS is a longitudinal survey that follows individuals every two years. In order to evaluate the effects of the COVID-19 pandemic, we use the data from surveys of CFPS conducted in 2018 and 2020 (hereafter, CFPS 2018 and CFPS 2020, respectively), that is, the year before the COVID-19 pandemic and the year during the COVID-19 pandemic, respectively. CFPS 2018 and 2020 received ethical approval from the Peking University Institutional Review Board (IRB00001052-14010). The updated version of CFPS 2018 was published on 31 December 2020, and CFPS 2020 was published on 30 December 2021. Thus, the data in this study will be the most feasible way to explore the effects of the COVID-19 pandemic on the addictive behaviors of the general Chinese population.

### 2.2. Variables

The dependent variable “Smoking” is constructed through the question “Did you smoke cigarettes in the past month?”. If a respondent answered “Yes”, then “Smoking” equals 1 and 0 otherwise. As cannabis use in China is illegal, nationally representative surveys like CFPS do not have information about cannabis. Thus, we do not consider cannabis use in measuring smoking behavior. The other dependent variable “Drinking” is constructed through the question “Did you drink alcohol at least 3 times a week in the past month?”. If an individual answered “Yes”, then “Drinking” equals 1 and 0 otherwise.

Independent variables include COVID-19, Age, Gender, Married, Hukou, Education (Illiteracy, Primary school, Junior high school, Senior high school, and University or above), Self-reported health (SRH poor, SRH fair, SRH good, SRH very good, and SRH excellent), and Work. In addition, Drinking is controlled in the regression of Smoking, and Smoking is controlled in the regression of Drinking.

The variable “COVID-19” indicates whether an observation belongs to the survey during the COVID-19 pandemic. COVID-19 equals 1 if the observation belongs to CFPS 2020, and it equals 0 if the observation belongs to CFPS 2018. The variable “Gender” equals 1 if the individual is a male and 0 otherwise. The variable “Married” equals 1 if the individual is married and 0 otherwise. The variable “Hukou” indicates the hukou status of an individual. Hukou is a residential registry system in China, it records the information of individuals such as name, date of birth, relatives, marital status, permanent address and category of residence (urban or rural). Hukou equals 1 if the hukou of the individual is urban, and it equals 0 if the hukou of the individual is rural.

The variable “Illiteracy” indicates that an individual has not received any education. The variable “Primary school” indicates that an individual has received 1–6 years of education. The variable “Junior high school” indicates that an individual has received 7–9 years of education. The variable “Senior high school” indicates that an individual has received 10–12 years of education. The variable “University or above” indicates that an individual has received more than 13 years of education.

The variables about self-reported health (SRH) are constructed through the question “How would you rate your health status?”. The answers to this question include “Excellent”, “Very good”, “Good”, “Fair”, and “Poor”. For SRH poor, if an individual expresses an affirmative answer to the choice “Poor”, SRH poor equals 1 and 0 otherwise. SRH fair, SRH good, SRH very good, and SRH excellent are constructed in a similar way.

Finally, the variable “Work” is constructed through the question “Including agricultural work, waged job, self-employment, and private business (housework and unpaid help do not count), have you worked for at least one hour last week?”. If an individual answered “Yes”, Work equals 1 and 0 otherwise.

### 2.3. Statistical Analyses

In statistical analyses, we decompose the sample into three age groups, that is, ages between 16 and 39, ages between 40 and 59 and ages above 60 (hereafter, Age 16–39, Age 40–59 and Age 60+, respectively). Age 60+ represents retired individuals. Age 16–39 and Age 40–59 represent individuals who are active in the labor market. Further, Age 16–39 captures individuals in the fast-rising stage of their careers, while Age 40–59 captures individuals in the relatively stable stage of their careers.

In the description of sample characteristics, we use the count and percentage to describe categorical variables, and we use the mean and standard deviation to describe the continuous variable. In addition, we use the bootstrap method to calculate the confidence interval of the difference in the mean of addictive behaviors.

In the regression analysis, we use logit models to analyze the effects of the COVID-19 pandemic on smoking and drinking behaviors. We estimate the model for three age groups separately in order to examine the heterogeneous effects of the COVID-19 pandemic on individuals of different ages. In addition to the above-mentioned independent variables, we also add the square of Age in order to control the nonlinear effect of age. Finally, we show the estimation results by reporting the odds ratio (hereafter OR) and 95% confidence interval (hereafter CI). We also cluster standard errors at the individual level. The analyses in this study are performed in Stata 15.

## 3. Results

We analyze a sample of individuals over 16 years of age in China, which consists of 14,468 individuals and 28,936 observations. Sample characteristics are shown separately for three age groups (Table 1). The percentage of smoking behaviors increases with age (27.59% for Age 16–39; 29.3% for Age 40–59; 30.67% for Age 60+). The pattern of drinking behavior is analogous to that of smoking behavior but has a steeper trend of increase (10.85% for Age 16–39; 15.83% for Age 40–59; 17.76% for Age 60+). The percentages of males and females are almost equal (Age 16–39: 48.14%, for males vs. 51.86%, for females; Age 40–59: 48.48%, for males vs. 51.52%, for females; Age 60+: 55.06%, for males vs. 44.94%, for females), indicating that our sample is balanced. The majority of individuals are married, especially individuals aged between 40 and 59 and above 60 (72.06% for Age 16–39; 98.57% for Age 40–59; 99.12% for Age 60+). The levels of education of different age groups are consistent with the reality in China that with the rapid development of the economy and education, younger individuals have higher percentages of receiving a university education. The self-reported health condition of individuals decreases with age. We found that 87.09% of individuals aged between 16 and 39 reported “Excellent”, “Very good”, or “Good”, while such a proportion decreases to 69.39% for Age 40–59 and 57.34% for Age 60+. Finally, around four-fifths of individuals aged below 60 worked for at least one hour last week. However, for retired individuals (Age 60+), only 49.71% of individuals in this group worked for at least one hour last week.

Panel A of Table 2 shows the individual changes in smoking and drinking behaviors before and during the COVID-19 pandemic. 5.72% of individuals change their smoking behaviors, where 2.16% of individuals began to smoke during the pandemic, and 3.56% of individuals quit smoking. 11.66% of individuals change their drinking behaviors, where 4.82% of individuals began to drink during the pandemic, and 6.84% of individuals quit drinking. Panel B of Table 2 gives the mean differences of Smoking and Drinking before and during the COVID-19 pandemic. The mean of Smoking is greater before the pandemic than during the pandemic (mean difference 0.0140, 95% bootstrap CI 0.0031–0.0248, statistically significant at 5% significance level). In addition, the pattern for Drinking is analogous to Smoking (0.0201, 0.0122–0.0280).

**Figure 1:**
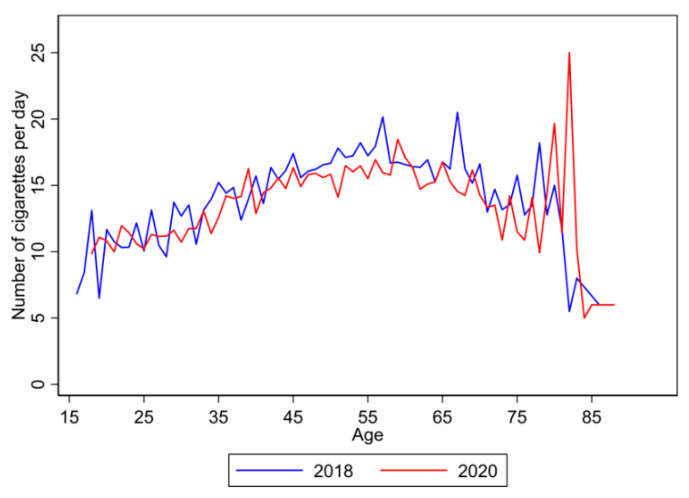
Comparison of number of cigarettes consumed per day among smokers between 2018 and 2020.

By extracting information from a question in CFPS, that is, “How many cigarettes do you smoke per day?”, Figure 1 shows the comparison of average numbers of cigarettes consumed per day among smokers between 2018 and 2020. In Figure 1, there is clear evidence that smokers in Age 40–59 consumed fewer number of cigarettes per day during the pandemic than before the pandemic.

Table 3 and Table 4 show the logit regression results for the effects of the COVID-19 pandemic on smoking and drinking behaviors, respectively. The odds ratio (OR) of COVID-19 is the parameter of interest. As shown in Table 3, for the age group “Age 16–39”, the OR of COVID-19 is statistically significant at a 10% significance level and shows that individuals aged between 16 and 39 smoke more during the pandemic than before the pandemic (1.068, 0.993–1.148). In this regression, Gender (Male = 1), University or above and Drinking are statistically significant at a 1% significance level. Specifically, a male is more likely to smoke (OR 117.803, 95% CI 82.382–168.453), and individuals with an education level of university or above are less likely to smoke (0.273, 0.152–0.490). Individuals with drinking behaviors are more likely to smoke (2.070, 1.714–2.500). The OR of Hukou (Urban = 1) demonstrates that urban residents are more likely to smoke (1.257, 1.035–1.526).

For “Age 40–59” the OR of COVID-19 is statistically significant at a 1% significance level and shows that individuals with ages between 40 and 59 smoke less frequently during the pandemic than before the pandemic (0.880, 0.835–0.927). Individuals with good self-reported health are more likely to smoke (1.201, 1.004–1.437).

For “Age 60+”, analogously, the OR of COVID-19 shows that individuals aged above 60 smoke less frequently during the pandemic than before the pandemic (0.898, 0.842–0.959). The OR of Work implies that individuals who still worked after retirement are more likely to smoke (1.190, 1.017–1.391).

In Table 4, the ORs of COVID-19 in all three age groups are statistically significant and less than 1, indicating that individuals at any age drink less during the pandemic than before the pandemic (0.710, 0.626–0.804 for Age 16–39; 0.852, 0.788–0.920 for Age 40–59; 0.868, 0.789–0.955 for Age 60+). Further, the ORs of Married in Age 16–39 and Age 40–59 are statistically significant and greater than 1, implying that married individuals who are active in the labor market are more likely to drink (1.409, 1.103–1.801 for Age 16–39; 1.637, 0.997–2.686 for Age 40–59). Finally, in all age groups, the ORs of Smoking are statistically significant at a 1% significance level and greater than 1, indicating that individuals with smoking behaviors are more likely to drink (2.084, 1.726–2.516 for Age 16–39; 1.519, 1.325–1.740 for Age 40–59; 1.399, 1.182–1.657 for Age 60+).

## 4. Discussion

This study uses nationally representative longitudinal data and explores the effects of the COVID-19 pandemic on the smoking and drinking behaviors of the general Chinese population. One of the main characteristics of the analysis is to differentiate individuals of different ages. Our results show that the COVID-19 pandemic reduces the smoking behavior of individuals above 40 years of age, and that it reduces the drinking behavior of individuals above 16 years of age. However, the pandemic increases the smoking behavior of individuals between 16 and 39 years of age.

In terms of smoking behavior, as COVID-19 is a respiratory system disease and particularly dangerous for older adults, compared to individuals below 40 years of age, individuals above 40 years of age may worry more about whether smoking will increase the infection risks of COVID-19 and thus smoke less after the outbreak of the pandemic. In addition, compared to individuals above 40 years of age, individuals below 40 years of age are likely in the fast-rising stage of their careers and may normally have heavier working and economic pressures. Thus, especially under the COVID-19 pandemic, they may have a stronger need to smoke in order to relieve pressure. In such a circumstance, for young adults, it is possible that the needs for smoking in the work and daily life during the pandemic outweigh the increased perception of infection risks due to smoking. As a result, the COVID-19 pandemic may increase the smoking behavior of young adults.

In terms of drinking behavior, in many regions of China, drinking alcohol is one of the major means of building and maintaining social networks. During the pandemic, the less frequent social activities due to lockdowns and working/staying at home decrease the needs for drinking. Even though deteriorated mental health during the pandemic is conducive to the increase in drinking, the effects of reduced social activities may dominate. Thus, we observe a clear pattern that the COVID-19 pandemic reduces the drinking behaviors of all adults. In fact, an interesting message delivered from our results is that although the COVID-19 pandemic is a health crisis, it may surprisingly ameliorate the addictive behaviors of a large proportion of the general population.

Our results also indicate that a higher education level may result in less use of tobacco. Consequently, developing education systems and encouraging people to receive more education can be a useful tool to reduce addictive behaviors in a country. Furthermore, as individuals who believe that they have good health may smoke and drink more, some public policies and propaganda work can be necessary to disseminate the message about the harm of heavy smoking and binge drinking.

This study also has several limitations. First, the frequency of smoking was not used to measure smoking behavior. In fact, in the questionnaires of CFPS, there is a question about the frequency of smoking: “How many cigarettes do you smoke per day?”. Nevertheless, the number of responses to this question is too limited. Thus, although the frequency of smoking is a better measure for smoking behavior, we cannot use it in this study.

Second, as there does not exist a question for the amount of alcohol consumption in the questionnaires of CFPS, the amount of alcohol consumption was not used to measure the drinking behavior. In fact, even if an individual drinks alcohol at least 3 times a week, that is, the measure of drinking behavior in this study, as long as the amount of alcohol he/she drinks every time is sufficiently small, it may not be accurate to state that he/she has an addictive behavior in terms of drinking. Therefore, using more proxies to measure addictive behaviors can be an important avenue for future research.

In addition, given that the objective of this study is to examine the effects of the pandemic, we did not fully characterize the relationship and the relative importance among the risk factors of addictive behaviors. In future research, methods such as the classification and regressions trees (CART) can be used to achieve further exploration.

Finally, controlling more sociodemographic factors and risk attitudes (such as risk-loving, risk-neutral and risk-averse preferences) of individuals in the regression analysis is also highly valuable.

## 5. Conclusions

Analyses using nationally representative longitudinal data show that except for the smoking behavior of young adults, the COVID-19 pandemic reduces the addictive behaviors (smoking and drinking) of the general population in China. These results may be closely related to the characteristics of COVID-19 (that is, a respiratory system disease), the working and economic pressures of young Chinese and the role of drinking alcohol in building and maintaining social networks in China.

## Figures and Tables

**Table 1:** Sample characteristics.

Age Groups	Age 16–39		Age 40–59		Age 60+	
Variables	N	Percentage or Mean (SD)	N	Percentage or Mean (SD)	N	Percentage or Mean (SD)
**Age**	9051	30.76 (5.18)	12,814	49.87 (5.32)	7071	67.10 (5.24)
**Smoking**						
Yes	2497	27.59	3754	29.3	2169	30.67
No	6554	72.41	9060	70.7	4902	69.33
**Drinking**						
Yes	982	10.85	2029	15.83	1256	17.76
No	8069	89.15	10,785	84.17	5815	82.24
**COVID-19**						
Yes	4295	47.45	6436	50.23	3737	52.85
No	4756	52.55	6378	49.77	3334	47.15
**Gender**						
Male	4357	48.14	6212	48.48	3893	55.06
Female	4694	51.86	6602	51.52	3178	44.94
**Married**						
Yes	6522	72.06	12631	98.57	7009	99.12
No	2529	27.94	183	1.43	62	0.88
**H** **ukou**						
Urban	2346	25.92	3311	25.84	2241	31.69
Rural	6705	74.08	9503	74.16	4830	68.31
**Education**						
Illiteracy	287	3.17	1838	14.34	2095	29.63
Primary school	599	6.62	2950	23.02	1878	26.56
Junior high school	3349	37	5170	40.35	1886	26.67
Senior high school	1771	19.57	1898	14.81	992	14.03
University or above	3045	33.64	958	7.48	220	3.11
**Self-reported health (SRH)**						
Poor	537	5.93	2164	16.89	1818	25.71
Fair	631	6.97	1757	13.71	1198	16.94
Good	4428	48.92	5599	43.69	2659	37.6
Very good	1911	21.11	1592	12.42	694	9.81
Excellent	1544	17.06	1702	13.28	702	9.93
**Work**						
Yes	7340	81.1	10,344	80.72	3515	49.71
No	1711	18.9	2470	19.28	3556	50.29
**N**	9051		12,814		7071	

SD = standard deviation.

**Table 2:** Changes in smoking and drinking behaviors before and during the COVID-19 pandemic.

Panel A			(2018, 2020)				
			(No, No)		(No, Yes)	(Yes, Yes)	(Yes, No)
**Smoking**	**N**		9844		313	3796	515
	**Percentage**		68.04		2.16	26.24	3.56
**Drinking**	**N**		11,491		698	1290	989
	**Percentage**		79.42		4.82	8.92	6.84
**Panel B**	**2018**		**2020**				
	**Mean**	**SD**	**Mean**	**SD**	**Mean Difference**	**95% Bootstrap CI**	***p*-Value**
**Smoking**	0.2980	0.4574	0.2840	0.4510	0.0140	(0.0031, 0.0248)	0.011
**Drinking**	0.1575	0.3643	0.1374	0.3443	0.0201	(0.0122, 0.0280)	<0.001

SD = standard deviation. CI = confidence interval.

**Table 3:** Logit regression results for the effects of COVID-19 pandemic on smoking behavior.

	Age 16–39		Age 40–59		Age 60+	
	OR	95% CI	OR	95% CI	OR	95% CI
**COVID-19**						
No	(ref)		(ref)		(ref)	
Yes	1.068 *	0.993–1.148	0.880 ***	0.835–0.927	0.898 ***	0.842–0.959
**Age**	1.150 *	0.995–1.329	0.794 **	0.641–0.983	1.004	0.758–1.332
**Age^2^**	0.998 **	0.995–1.000	1.002 **	1.000–1.005	1.000	0.998–1.002
**Gender**						
Female	(ref)		(ref)		(ref)	
Male	117.803 ***	82.382–168.453	63.763 ***	50.412–80.650	21.055 ***	16.495–26.875
**Married**						
No	(ref)		(ref)		(ref)	
Yes	1.083	0.872–1.345	0.987	0.635–1.533	1.211	0.642–2.283
**H** **ukou**						
Rural	(ref)		(ref)		(ref)	
Urban	1.257 **	1.035–1.526	1.052	0.892–1.239	0.871	0.723–1.051
**Education**						
Illiteracy	(ref)		(ref)		(ref)	
Primary school	0.709	0.376–1.339	0.952	0.732–1.237	0.820 *	0.649–1.037
Junior high school	0.741	0.420–1.307	0.645 ***	0.505–0.823	0.762 **	0.606–0.958
Senior high school	0.687	0.384–1.227	0.619 ***	0.467–0.820	0.570 ***	0.430–0.754
University or above	0.273 ***	0.152–0.490	0.463 ***	0.329–0.653	0.524 ***	0.321–0.854
**Self-reported health (SRH)**						
Poor	(ref)		(ref)		(ref)	
Fair	1.266	0.855–1.874	1.036	0.837–1.282	1.074	0.870–1.325
Good	1.256	0.910–1.734	1.201 **	1.004–1.437	1.164	0.967–1.401
Very good	1.406 **	1.000–1.975	1.152	0.926–1.433	1.039	0.799–1.351
Excellent	1.425 **	1.006–2.017	1.004	0.810–1.244	1.291 *	0.999–1.668
**Work**						
No	(ref)		(ref)		(ref)	
Yes	1.101	0.856–1.416	0.962	0.806–1.149	1.190 **	1.017–1.391
**Drinking**						
No	(ref)		(ref)		(ref)	
Yes	2.070 ***	1.714–2.500	1.532 ***	1.337–1.756	1.402 ***	1.184–1.660
**Constant**	0.002 ***	0.000–0.015	7.091	0.037–1353.468	0.151	0.000–2515.675
**N**	9051		12,814		7071	

*** *p* < 0.01, ** *p* < 0.05, * *p* < 0.1. OR = odds ratio. CI = confidence interval. SRH = self-reported health. In the estimation, we cluster standard errors at the individual level.

**Table 4:** Logit regression results for the effects of COVID-19 pandemic on drinking behavior.

	Age 16–39		Age 40–59		Age 60+	
	OR	95% CI	OR	95% CI	OR	95% CI
**COVID-19**						
No	(ref)		(ref)		(ref)	
Yes	0.710 ***	0.626–0.804	0.852 ***	0.788–0.920	0.868 ***	0.789–0.955
**Age**	0.968	0.824–1.136	0.921	0.748–1.134	1.009	0.747–1.363
**Age^2^**	1.001	0.998–1.004	1.001	0.999–1.003	1.000	0.998–1.002
**Gender**						
Female	(ref)		(ref)		(ref)	
Male	9.516 ***	7.002–12.933	10.398 ***	8.474–12.759	9.656 ***	7.457–12.505
**Married**						
No	(ref)		(ref)		(ref)	
Yes	1.409 ***	1.103–1.801	1.637 *	0.997–2.686	0.797	0.393–1.617
**Hukou**						
Rural	(ref)		(ref)		(ref)	
Urban	0.832	0.668–1.036	1.168 *	0.996–1.370	0.888	0.726–1.086
**Education**						
Illiteracy	(ref)		(ref)		(ref)	
Primary school	2.166 **	1.102–4.260	0.892	0.695–1.144	0.913	0.726–1.149
Junior high school	2.412 ***	1.299–4.477	1.090	0.866–1.374	0.902	0.715–1.139
Senior high school	1.976 **	1.046–3.735	0.961	0.732–1.263	0.690 **	0.512–0.930
University or above	1.619	0.850–3.086	0.622 ***	0.441–0.878	1.096	0.679–1.769
**Self-reported health (SRH)**						
Poor	(ref)		(ref)		(ref)	
Fair	1.116	0.744–1.676	1.698 ***	1.345–2.143	1.951 ***	1.532–2.485
Good	0.849	0.606–1.191	1.642 ***	1.344–2.006	1.967 ***	1.588–2.438
Very good	0.811	0.566–1.162	1.925 ***	1.522–2.433	2.109 ***	1.596–2.786
Excellent	1.146	0.800–1.640	2.126 ***	1.687–2.680	2.116 ***	1.615–2.773
**Work**						
No	(ref)		(ref)		(ref)	
Yes	1.161	0.885–1.521	1.223 **	1.022–1.464	1.230 **	1.034–1.462
**Smoking**						
No	(ref)		(ref)		(ref)	
Yes	2.084 ***	1.726–2.516	1.519 ***	1.325–1.740	1.399 ***	1.182–1.657
**Constant**	0.008 ***	0.001–0.096	0.055	0.000–9.435	0.025	0.000–815.180
**N**	9051		12,814		7071	

*** *p* < 0.01, ** *p* < 0.05, * *p* < 0.1. OR = odds ratio. CI = confidence interval. SRH = self-reported health. In the estimation, we cluster standard errors at the individual level.

## Data Availability

The data is available at http://www.isss.pku.edu.cn/cfps/index.htm (accessed on 3 March 2022).
